# Integration of clinical and transcriptomics reveals programming of the lipid metabolism in gastric cancer

**DOI:** 10.1186/s12885-022-10017-4

**Published:** 2022-09-06

**Authors:** Yanyan Li, Jungang Zhao, Renpin Chen, Shengwei Chen, Yilun Xu, Weiyang Cai

**Affiliations:** 1 Department of Ultrasound, The Second Affiliated Hospital and Yuying Children’s Hospital of Wenzhou Medical University, Wenzhou, Zhejiang Province China; 2 Department of Hepatobiliary Surgery, The First Affiliated Hospital of Wenzhou Medical University, Wenzhou, China; 3 Department of Gastroenterology, The First Affiliated Hospital of Wenzhou Medical University, Wenzhou, China; 4 Department of Urology, Yuhuan People’s Hospital, Wenzhou, China

**Keywords:** Lipid metabolism, Multi-omics, Gene signature, ACLY

## Abstract

Lipid metabolism has a profound impact on gastric cancer (GC) progression and is a newly targetable vulnerability for cancer therapy. Given the importance of lipids in cancer cellular processes, in this study we employed lipidomic clinical and transcriptomic data to connect the variations of lipid metabolism changes of GC. We constructed a clinical nomogram based on the lipid factors and other clinical items. Then by using multi-omics techniques, we established a lipid-related gene signature for individualized prognosis prediction in patients with GC. Moreover, a total of 1357 GC cases were then applied to evaluate the robustness of this model. WGCNA was used to identify co-expression modules and enriched genes associated with GC lipid metabolism. The role of key genes *ACLY* in GC was further investigated. The prognostic value of the lipgenesis signature was analyzed using Cox regression model, and clinical nomogram was established. Among them, we observed overexpression of *ACLY* significantly increased the levels of intracellular free fatty acid and triglyceride, and activated *AKT/mTOR* pathway to promote cancer development. In conclusion, our findings revealed that GC exhibited a reprogramming of lipid metabolism in association with an altered expression of associated genes. Among them, *ACLY* significantly promoted GC lipid metabolism and increased cancer cell proliferation, suggesting that this pathway can be targetable as a metabolic vulnerability in future GC therapy.

**Supplementary Information:**

The online version contains supplementary material available at 10.1186/s12885-022-10017-4.

## Summary

1. Combined clinical and pathological data, we constructed a GC clinical nomogram. 2. A lipid-related gene signature was established for individualized prognosis prediction. 3. *ACLY* significantly promoted GC lipid metabolism and increased cancer cell proliferation.

## Introduction

Gastric carcinoma (GC) is commonly known as the second most cancers worldwide, which approximately accounts for 900,000 total cases and 700,000 deaths globally per annum, and the overall survival (OS) for GC patients diagnosed with metastatic still less than 1 year [1]. Hyperlipemia is a significant global health problem and regarded as a conspicuous risk factor for human cancers, specially for gastroenteric tumors, by recent statistics conducted by the International Agency for Research on Cancer [2, 3]. Lipids are classified into eight types basing on the presence of ketoacyl and isoprene groups, including fatty acids (FAs), glycerolipids, glycerophospholipids, sphingolipids, sterol lipids, prenol lipids, saccharolipids and polyketide. Epidemiological studies demonstrated that high fat diet and obesity increase the risk of GC, with obesity-persistence increase the risk of cancer in a does-response manner [4]. However, the association between lipid metabolism and the pathological development of gastric cancer remained confusing, and the relationship between lipid metabolism and cancer prognosis is still not explored.

The gastrointestinal tract is an important organ for food digestion and nutrient absorption, with the supply for cellular metabolism. Lipgenesis, including de novo fatty acid synthesis and cholesterol biosynthesis, ketone body metabolism, fatty acids synthesis (FAS), fatty acids oxidation (FAO), cholesterol metabolism pre-cursor for eicosanoid synthesis, which provides cancer cells with adequate energy supply. Cancer cells being in a nutrient-deficient environment, change their normal metabolism state for acquiring energy and building new biomass [5]. In case of nutrient deprivation, fatty acids released from lipid drops (LDs) are important energy source via mitochondria β-oxidation and Kreb’s cycle. More importantly, lipid metabolism is known not only functioned in orthodox energy supply and membrane components, but also involved in numerous cellular processes, such as cell signaling, inflammation maturation, storage, turnover of proteins, cell proliferation, cytoskeleton, cell polarity, signaling molecules, post-translational modifications, angiogenesis differentiation and so on [6, 7]. Thus, lipid is gradually recognized as a prominent characteristic in a variety of cancers and attracts increasing attention.

With the gradually deep understanding of the extensive roles of lipid metabolism in GC pathogenesis, the specific mechanisms of cancer cells have been exploited. Our analysis integrated clinical and lipidomics-transcriptomics data revealed that GC exhibited a reprogramming of lipid metabolism in association with an altered expression of associated genes. Our findings also highlighted the notion that *ACLY* may be a promising therapeutic target in GC and provides evidences for uncovering the link between lipodystrophy and tumor development.

## Materials and methods

### Patients and study design

This study was designed as a retrospective cohort study, which utilized data from the First Affiliated Hospital of Wenzhou Medical University and public databases and Gene Expression Omnibus (GEO) datasets. Selected GC patients were histopathologically diagnosed with primary GC and then received surgical treatment with or without regular chemotherapy. Finally, a total of 458 patients were enrolled in the study. Hyperlipoidemia was defined as conform to more than one criteria: 1) Total cholesterol (TC) than or equal to 5.17 mmol/l; 2) Triglyceride (TG) than or equal to 1.70 mmol/l; 3) High-density lipoprotein (HDL) less or equal to 1.16 mmol/l; 4) Low-density lipoprotein white (LDL) than or equal to 3.10 mmol/l. The clinicopathological characteristics of enrolled patients were listed in the Table 1.

**Table 1:** Clinicopathological characteristics of gastric cancer patients grouped by lipid index

Characteristics	TC		TG		HDL		LDL	
	< 5.1	≥5.1	< 1.7	≥1.7	≥1.42	< 1.42	< 3.1	≥3.1
**Sex**		0.159		**< 0.001**		**0.003**		**0.003**
Male	64	39	76	89	85	18	63	40
Female	272	83	266	27	240	115	269	86
**T stage**		**0.001**		**0.004**		**< 0.001**		**0.001**
T1	52	38	55	35	74	16	50	40
T2	47	19	50	16	53	13	49	17
T3	44	14	41	17	46	12	45	13
T4	193	51	196	48	152	92	188	56
**N stage**		**0.016**		**0.001**		0.463		**0.013**
N0	127	63	125	65	139	51	125	65
N1	55	20	63	12	55	20	54	21
N2	154	39	154	39	131	62	153	40
**M stage**		0.159		**0.041**		0.578		0.725
M0	306	116	310	112	298	124	305	117
M1	30	6	32	4	27	9	27	9
**Stage**		**0.005**		**0.002**		**< 0.001**		**0.043**
1	75	43	74	44	98	20	73	45
2	87	22	50	16	52	14	47	19
3	187	51	186	52	148	90	185	53
4	30	6	32	4	27	9	27	9
**Chemotherapy**		0.899		**< 0.001**		**0.041**		0.280
No	130	48	122	56	136	42	124	54
Yes	206	74	220	60	189	91	208	72
**BMI**		**0.034**		0.099		0.889		0.064
**< 18.5**	35	5	35	6	28	12	34	6
**18.5–25**	301	117	307	110	297	121	298	120

This study also utilized data from public database. We retrospectively selected GC gene expression and its clinicopathological data from the TCGA and Gene Expression Omnibus datasets. Raw GEO files were downloaded from GEO (https://www.ncbi.nlm.nih.gov/geo/). Raw microarray data Affymetrix were downloaded and normalized using the limma package. Chips were summarised, together with accession numbers. In total, 1488 GC and 448 normal cases were acceptable for subsequent meta-analysis in Table S1. The workflow of the paper was showed in the Fig. S1.

### Construction clinical nomogram

The OS and PFS clinical nomograms were constructed based on the main prognostic factors to predict 1-, 3- and 5-year survival of each GC patients. A multivariable logistic regression analysis was applied to build nomogram. The nomogram was generated using ggplot packages together with R software. The survival analysis was conducted using rms, survivalROC, survcomp and survival package. Hazard ratios (HRs) and 95% confidence intervals (CIs) were recorded.

### Establishment of the LASSO regression model and calculation of lipodystrophy risk score

We used the GSEA program to derive the enrichment scores of each lipid-metabolism Gene sets. The concrete gene lists of each lipid metabolism enrichment KEGG terms were listed in the Table 2. A lipid score was calculated for each patient via weighted by their LASSO Cox coefficients. Moreover, GSE15459, GSE26253, GSE62254 and GSE84437, which contain concrete survival information, were further employed to confirm the prognostic power of gene signature. The lipid score of each patient was calculated with the expression level and its relative coefficient. On the basis of the median score as the cut-off setline, these patients were divided into high- and low-subgroups. Log-rank test was performed to calculate the corresponding hazard ratios (HRs) and 95% confidence interval (CI).

**Table 2:** The specific markers for lipid-related pathways

KEGG	Gene
Unsaturated fatty acids	ACOT2, ACOT7, ACOT4, HACD2, ACAA1, HADHA, FADS1, ACOX1, HSD17B12, ELOVL2, YOD1, PECR, BAAT, ELOVL5, SCD, ACOT1, ELOVL6, ACOX3, HACD1, FADS2
Fatty acid metabolism	TECR, ACAA2, ECI2, ADH1A, ADH1B, ADH1C, CPT1C, ADH4, ADH5, ADH6, ADH7, CPT1A, CPT1B, CPT2, CYP4A11, ECI1, ECHS1, EHHADH, ALDH2, ACSL1, ACSL3, ACSL4, ALDH1B1, ALDH9A1, ALDH3A2, ACSL6, GCDH, CYP4A22, ACAA1, HADHA, HADHB, HADH, ACADL, ACADM, ACADS, ACADSB, ACADVL, ACAT1, ACAT2, ALDH7A1, ACOX1, ACSL5, ACOX
Steroid biosynthesis	CEL, EBP, CYP27B1, CYP51A1, DHCR7, DHCR24, FDFT1, LIPA, LSS, NSDHL, HSD17B7, MSMO1, SC5D, SOAT1, SQLE, TM7SF2, SOAT2,
Ether lipid metabolism	PLA2G4B, PLA2G4E, ENPP6, LPCAT4, PLA2G2D, PLA2G2E, PLA2G2C, PAFAH1B1, PLA2G3, PAFAH1B2, PAFAH1B3, PAFAH2, ENPP2, PLA2G1B, PLA2G2A, PLA2G5, PLD1, PLD2, LPCAT2, CHPT1, PLA2G2F, PLA2G7, LPCAT1, PLA2G12A, PLA2G6, PLA2G10, PLA2G12B, AGPS, PLPP1, PLPP2, PLPP3
Glycosphingolipid biosynthesis lacto and neolacto serie	B3GALT5, B3GNT3, ST3GAL6, B3GNT2, FUT9, B4GAT1, FUT1, FUT2, FUT3, FUT4, FUT5, FUT6, FUT7, B4GALT1, ABO, ST3GAL4, ST3GAL3, ST8SIA1, B3GN4, B3GNT5, B4GALT4, B2GAL3, B4GALT2, B3GALT1

### Co-expression gene network based on RNA-seq data and functional analysis

The Weighted correlation network analysis (WGCNA) was used to identify important co-expression modules and their enriched genes associated with GC lipid metabolism [8]. The co-expression network was constructed by the R package WGCNA. The connectivity degree of each node of the network was calculated by STRING database and reconstructed via Cytoscape software.

### Colony formation and Transwell migration assay

The number of 1 × 10^3^ BGC823 and SGC7901 were inoculated in six-well plates and incubated at 37 °C for 5 days. Cell colonies were finally fixed with 4% paraformaldehyde formaldehyde (Solarbio, Beijing, China) followed by staining with crystal violet (Sigma-Aldrich).

### Western blot, RT-PCR and antibodies

An equal amount of proteins was subjected to SDS-PAGE. Proteins were transferred onto PVDF membranes, and the blots were incubated with the following different primary antibodies: Rabbit p-mTOR (S2448), p-AKT, β-actin from Cell Signaling Technology and IL6 from Proteintech.

### Quantification of free fatty acid and cholesterol

We prepared chloroform/methanol (2:1) for extracting lipids. The levels of free fatty acid and cholesterol were determined with EnzyChromTM free fatty acid and cholesterol kits (Bioassay Systems, Hayward, CA, USA).

### Immunohistochemistry

20 GC specimens were collected, of which 10 hyperlipemia and 10 non- hyperlipemia GC tissues. Two researchers evaluated the staining results independently and scored the intensities of immunostaining as: 0 (negative), 1 (weakly positive), 2 (moderately positive) and 3 (strongly positive). For IHC score, the percentage (0–100%) of stained tumor cells was multiplied by the intensity (0, 1, 2, or 3) to achieve a score between 0 and 300.

### Statistical analysis

R software and Stats were used for statistical analyses. Continuous variables were exhibited for means, medians, range, and standard deviation (SD) and compared using an independent t test or Wilcoxon test；Spearmen correlation coefficient was used for variable correlation; Chi-square test was used to analyze categorical variables. The log-rank survival test and the results were shown in the forest plot. All statistical tests were two-sided and *P* < 0.05 was considered statistically significant.

## Results

### Association of lipid biomarkers with clinicopathological features

According to our selection criteria, a total of 458 GC patients were included in this retrospective study. One hundred and three (22.5%) patients were female, and 355 (77.5%) were male; the median age was 64.3 (range, 29–87) years. About 38.9% of the patients only received surgery, while the others received both regular adjuvant postoperative therapy and surgery. The median follow-up for OS and PFS was 40.67 and 37.21 months respectively. Hyperlipemia is conferred as meeting one of the following items: the serum total TC, TG and LDL-C are increased and HDL-C is decreased. In our study, the proportions of patients with high- TC, TG, LDL-C and HDL-C were 26.64, 29.04, 25.33 and 27.51%, respectively. Totally, there were 288 patients diagnosed with hyperlipemia and 170 patients with non- hyperlipemia. Baseline clinicopathological characteristics were summarized in Table 1.

**Fig. 1:**
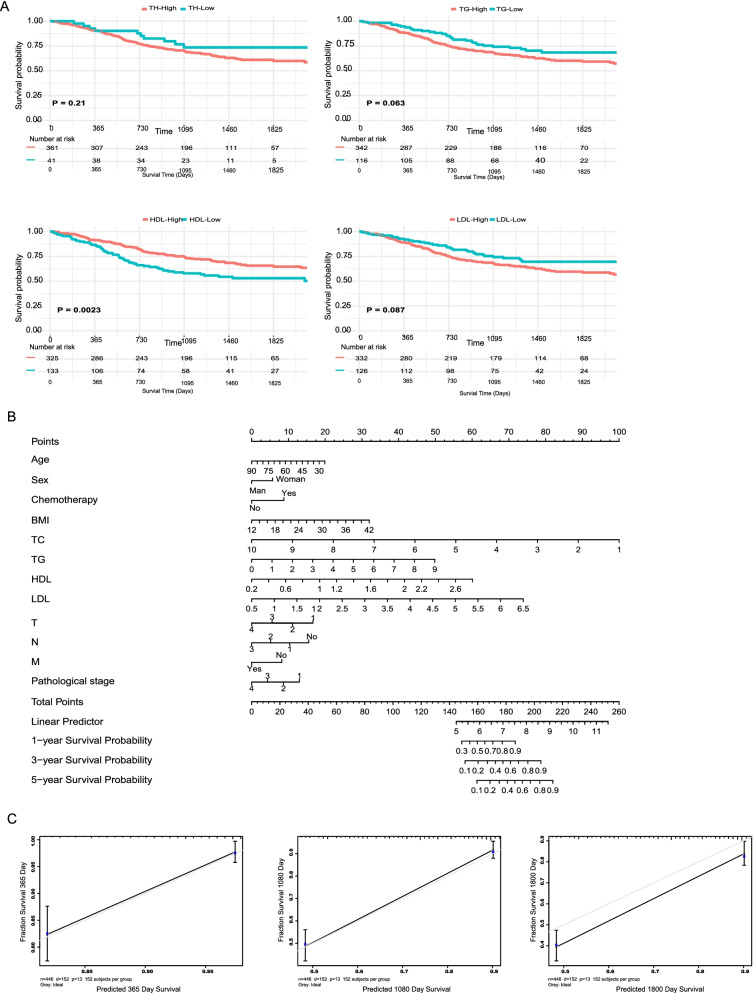
Kaplan-Meier curves for GC patients stratified by clinical lipid index. **A** Kaplan-Meier analysis of OS of Cholesterol, Triglyceride, HDL and LDL; **B** Nomogram developed by integrating lipid index and other clinical pathological parameters for predicting 1-, 3-, 5-year survival of GC patients; **C** Calibration curve for risk of 1-, 3-, 5-year survival of GC patients

The results of univariate Cox hazards analysis was presented in Fig. 1 and Fig. S2. The univariate analysis demonstrated that HDL, TC and LDL were significant associated with PFS (Fig. 1A, *P* < 0.05). Although some univariate Cox hazard results of lipid factors did not reach statistical significance, we saw a clear trend and called the result promising and worth further studies. An OS and PFS nomogram were constructed to predict 1-, 3- and 5-year overall survival (Fig. 1B) and PFS (Fig. S2B) of GC patients. Total scores were summations of each variable based on the intersection of the vertical line. As shown in Fig. 1B and Fig. S2B, TC and LDL contributed the most risk points, whereas the other clinical information contributed much less. By sum of the total points from all variables combined with the location at the total point, we could obtain the probabilities of survival outcomes of 1-year, 3-year, and 5-year. In addition, decision curve analysis showed that both the predictive accuracy of prognostic nomograms for OS and PFS (Fig. 1C and Fig. S2C).

### Identification of lipid metabolism signature in patients with GC

**Fig. 2:**
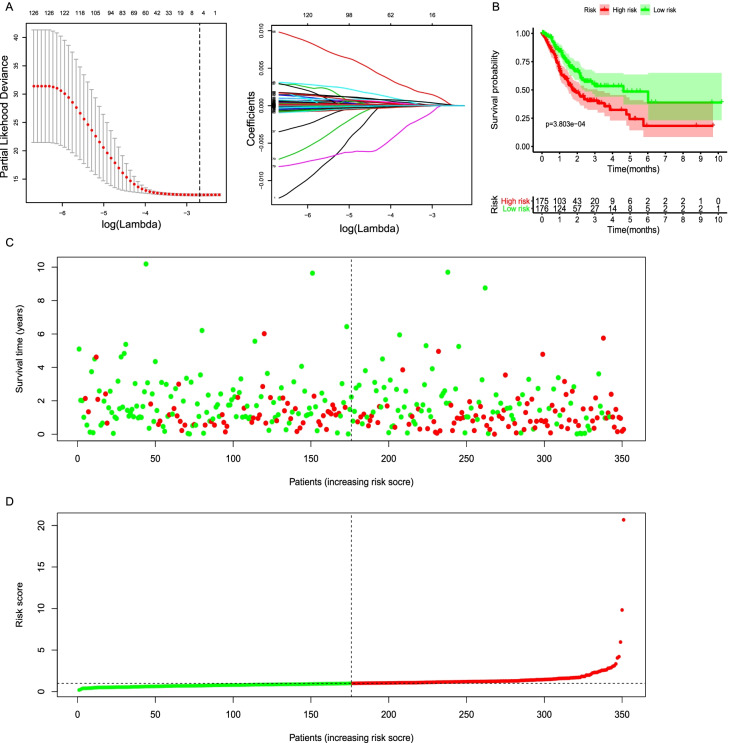
The distribution of gene lipidomics score in the TCGA GC cohort. **A** Feature selection with LASSO binary logistic regression model. The left part: The longitudinal solid line represents the partial likelihood deviation ±standard error and the longitudinal dotted line indicates that the best parameter is selected according to the minimum value (left) and 1-SE (right). Lambda is the tuning parameter. The right part: y axis represents Coefficients. Each curve in the graph corresponds to the value of each characteristic regression coefficient varying with the log (Lambda) value. **B** K-M survival curve of the low- and high- lipid score for TCGA GC patients; **C**-**D** The distributions of the lipid score and survival status for GC patients

Gene Set Enrichment Analysis (GSEA) is a computational method that defined set of genes shows statistically differences between two biological states [9]. The significantly enriched lipid-related pathways included unsaturated fatty acids, fatty acid metabolism, steroid biosynthesis, ether lipid metabolism and glycosphingolipid biosynthesis lacto and neolacto serie. A total of 133 lipid-relative genes were significantly enriched in the pathways related to lipid metabolism and gastric cancer (Table 2). LASSO Cox regression was performed to identify the most important features in terms of predicting the survival of GC patients (Figs. 2A). By forcing the sum of the absolute value of the regression coefficients to be less than a fixed value, certain coefficients were reduced to exactly zero, and the most prognostic features (*ACLY, ABCG4, ABCG1, FTO, ABCA2, IGFBP7*) were identified with relative regression coefficients. The prognostic risk score model was established with the following formula: lipid score = expression level of *ACLY* × 0.47 + expression level of *ABCG4*× 0.133 + expression level of *ABCG1* × 0.2238 + expression level of *FTO*× 0.274-expression level of *ABCA2* × 0.385 + expression level of *IGFBP7* × 0.210. All patients were divided into the high- and the low-lipid group using the median score as the cutoff line (Fig. 2B-D). K-M survival analysis showed that high risk group had significantly poorer OS than that of low (log-rank *P* < 0.001). In addition, following the univariate and multivariate analyses, the lipid signature also showed to be an independent prognostic factor in the GC cohorts (95%HR1.78–2.12, *P* < 0.001; 95%HR1.65–1.92 *P* < 0.001).

### Evaluation of the prognostic lipid metabolism signature in external validation cohorts

**Fig. 3:**
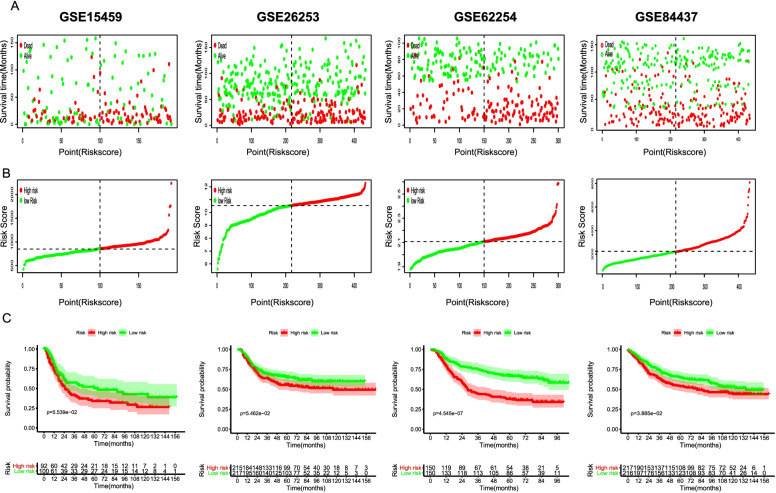
Validation the lipidomics signature in validation cohort. (A) Patient survival status and time distributed by lipid-score for each validation cohort; (B) the distribution of lipid score for each validation cohort; (C) K -M survival curve of the lipid-score for the OS time of each validation cohort. The dotted line indicates the individual inflection point of the risk score curve, by which the patients were categorized into low- and high-risk groups

To verify the accuracy of prognostic model identified by TCGA were also important in additional GC cases, we further selected eligible cohorts of GC cases from the GEO database (GSE15459, GSE26253, GSE62254 and GSE84437). As result, a total of 1357 GC cases were applied to evaluate the robustness of our model. Consistent with the results in the train cohort, the four survival analysis all showed that high lipid group had a worse prognosis than low one. The distribution of lipid-score, and survival information of patients were analyzed and showed in Fig. 3A-C. In brief, these external validation outcomes combination with prior studies demonstrated that our lipid signature were powerful enough to precisely discriminate high lipid score of GC patients.

### Functional annotation and WGNCA of GC patients

**Fig. 4:**
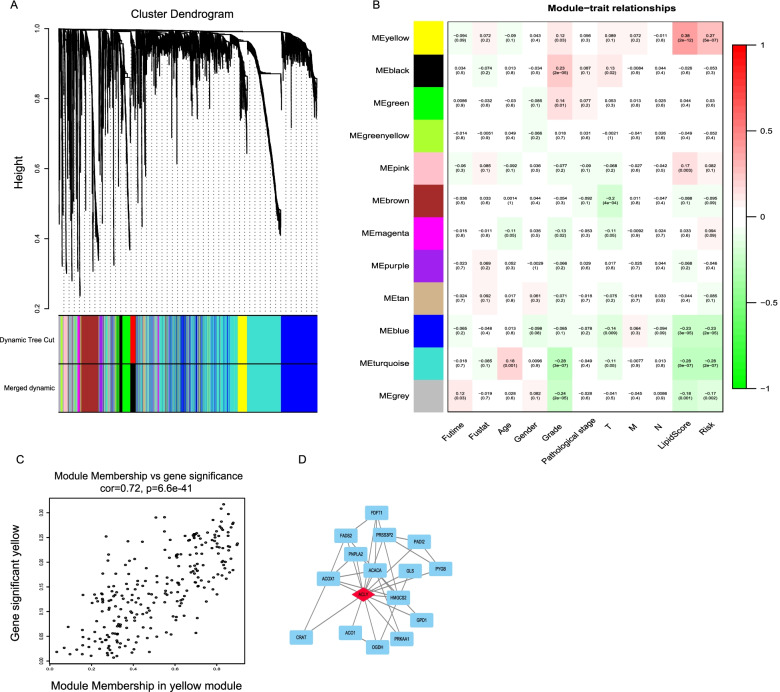
WGCNA identified lipid -related modules eigengenes. **A** Hierarchical clustering dendrogram of identified gene. **B** Heat map to show the correlation between module eigengenes and the clinical traits; The right color scale indicates the association. Red, positive associations; green, negative associations. The left color scale is corresponding to each module. **C** The correlation plot of gene significance in the yellow module; **D** Construction of the PPI network for top differentially expressed mRNAs in the yellow module

To further identify the underlying biological characteristics in the lipid metabolism signature, WGCNA was performed, and the correlation of lipid score and module membership were analyzed. The soft threshold selection is shown in Fig. 4A. The yellow module had a significant *p*-value with lipid signature (Fig. 4B). The association between module membership and gene significance for each gene in the yellow module is shown in Fig. 4C. To better annotate the module function, we singled out the 20 central genes in the co-expression network whose MM > 0.8. As shown in the Fig. 4D, *ACLY* is the hub gene in the GC lipid regulation.

### ACLY markedly elevated expression in the GC tissues

**Fig. 5:**
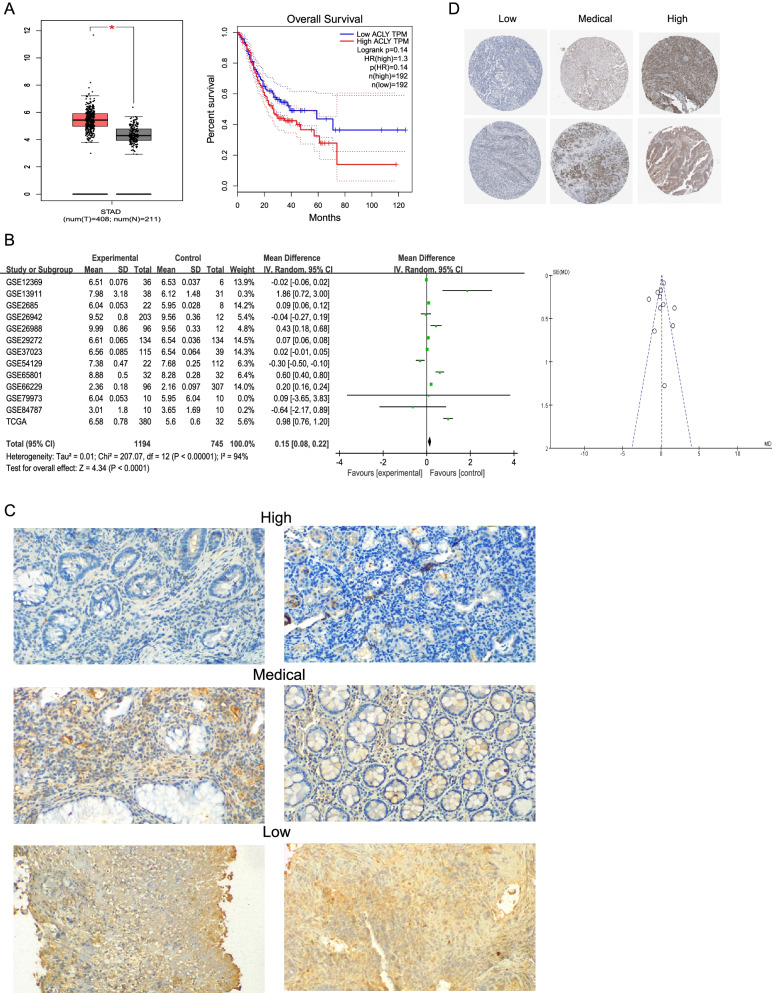
Expression and survival analysis of ACLY in GC. **A** The mRNA expression of ACLY in normal and GC tissues in the TCGA GC dataset. K-M OS curves based on the expression levels of ACLY; **B** Meta-analyze verified ACLY mRNA expression in 13 datasets; **C** ACLY immunostaining of representative images of GC patients with different IHC scores; (D) ACLY immunostaining of representative images of GC patients in HPA database

Genome data from TCGA suggested that *ACLY* gene is amplified in mostly GC cases (Fig. 5A). To further determine the *ACLY* mRNA expression in GC tissues, we examined the open GEO datasets which contain both GC specimens and normal specimens (Additional Table 1). In total, 1488 GC and 448 normal cases were enrolled in the subsequent meta analyses. Notably, the results showed that the *ACLY* mRNA expression exhibited a significantly increasing trend in group of GC specimens compared with normal specimens (Z = 4.34; *p* < 0.0001, Fig. 5B). These meta-analyze combination with prior outcomes mainfested that *ACLY* mRNA expression was significantly enhanced in GC tissues.

To evaluate the clinical significance of *ACLY* overexpression, we analyzed its protein expression by immunohistochemistry (IHC) in 30 GC patients. As shown in the Fig. 5C, GC patients with high *ACLY* expression were endowed with advanced pathological stage than those with low *ACLY* expression in cohorts. Essentially the same result was seen with HPA GC cohort (Fig. 5D). These results robustly demonstrate that *ACLY* was an independent prognostic predictor of poor survival in patients with GC.

### ACLY increased the expression levels of fatty acid synthesis enzymes and AKT/mTOR signaling

**Fig. 6:**
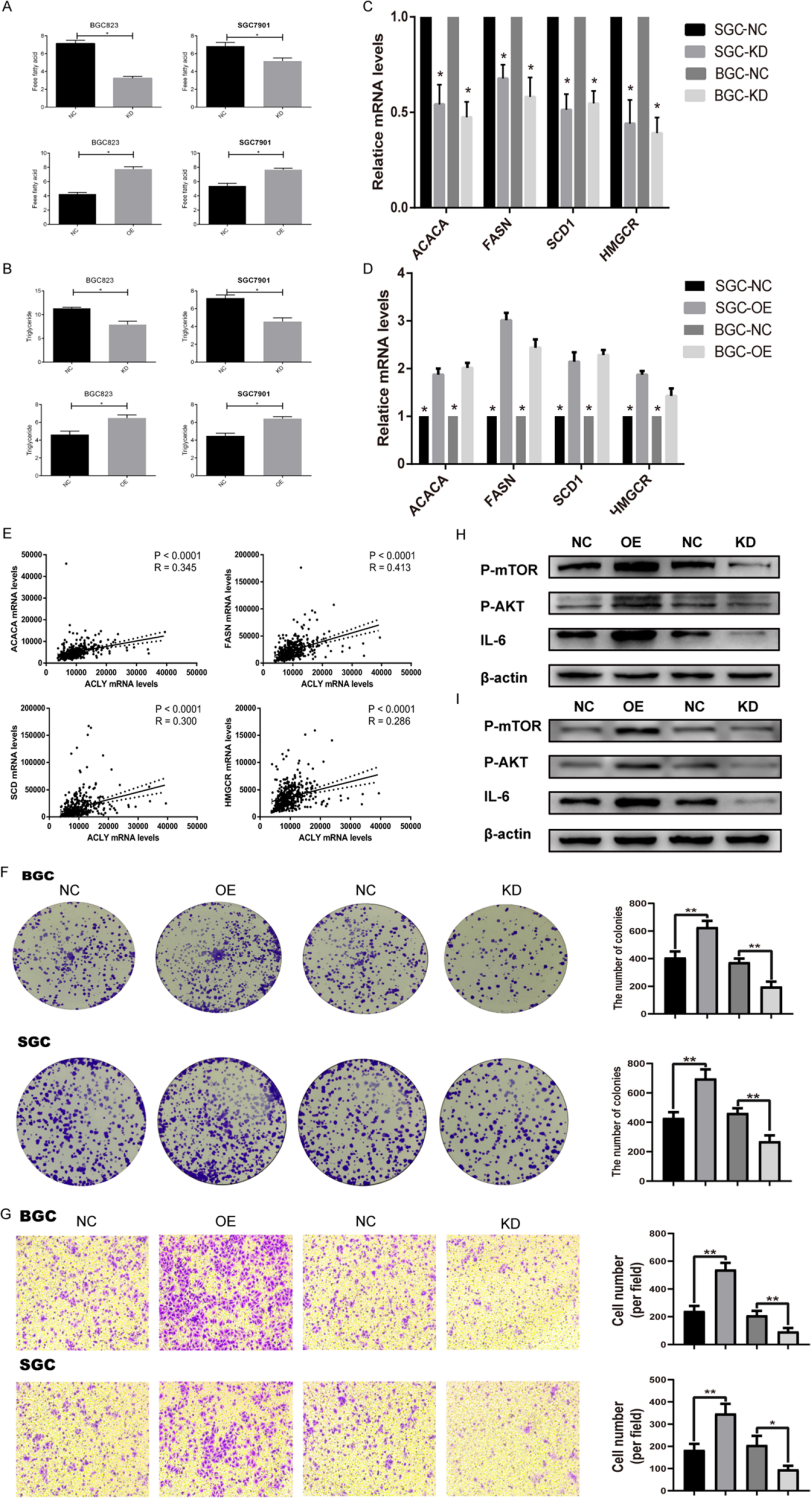
ACLY increased the expression level of fatty acid synthesis enzymes and AKT/mTOR signaling. Intracellular levels of free fatty acid (**A**) and triglyceride (**B**) with ACLY knocked-down or over-expressed; Quantitative RT-PCR analysis for mRNA levels of lipogenic enzymes with ACLY knocked-down (**C**) and over-expression (**D**); **E** Spearman correlation analysis of the mRNA expression levels of ACLY and lipogenic enzymes; Colony formation assay (**F**) and Transwell assay (**G**) in GC cells with ACLY knocked-down or over-expressed; BGC823 (**H**) and SGC7901 (**I**) expressing ectopic ACLY or vector were analyzed for mTOR signaling by immunoblotting

To study the role of *ACLY* in the lipid metabolism of GC cells, we designed to measure the changes of lipid content in gastric cancer cells with relative higher and lower *ACLY* expression. As shown in the Fig. 6A-B, overexpression of *ACLY* significantly increased the levels of intracellular free fatty acid and triglyceride, while knockdown of *ACLY* markedly reduced the levels of those lipids. We further investigated the expression levels of key molecules involved in fatty acid metabolism (*ACACA, FASN, SCD1, HMGCR*) in GC tissue when *ACLY* was knocked-down or over-expressed. As shown in the Fig. 6C-D, the expression of *ACLY* resulted in significantly change expression levels of those lipogenic enzymes. Thus, we inferred that *ACLY* increased de novo fatty acid synthesis and cholesterol biosynthesis in GC tissues. To provide further support, the expression levels of *ACLY* and lipogenic enzymes were determined in 380 GC tissue samples from TCGA. Spearman rank correlation analysis indicated significantly positive correlations between the expression levels of *ACLY* and lipogenic enzymes of ACACA (r = 0.345, *p* < 0.0001), FASN (r = 0.413, < 0.0001), SCD1 (r = 0.300, < 0.0001) and HMGCR (r = 0.286, < 0.0001) (Fig. 6E).

It is consensus viewed that lipids are a broad church of hydrophobic biomolecules that participate in a wide array of metabolic pathways, and can influence cancer cell biology via a range of multiple oncogenic signaling pathways. Considering the *mTOR* pathway is a master regulator of cell growth and metabolism in response to nutrient signals, particularly lipid. A key example is the well-defined influence of *PI3K-mTOR*, which activates multiple oncogenic signaling pathways, such as *AKT* signaling, during tumor progression [10]. Considering that *AKT/mTOR* pathway has been well established to play a central role in the regulation of cell lipid metabolism [11, 12], we hypothesized that *ACLY* overexpression may activate *AKT/mTOR* pathway to promote cancer development. As expected, ectopic expression of ACLY was sufficient to robustly promote GC cell migration and transwell compared with control cells (Fig. 6F and G). *ACLY* knockdown significantly decreased the phosphorylation levels of AKT and mTOR, whereas in inverse, indicating that *ACLY* activates AKT/ mTOR signaling in GC cells (Fig. 6H-I). Thus, we came up with an assumption that *ACLY* increased GC progression by activating *AKT/mTOR* signaling.

## Discussion

Hyperlipemia and its related complications are significant health problems with an estimated global prevalence of 3.9 million [13]. Research has shown that lipid metabolism may play a role in the development and progression of various malignancies. Higher LD accumulation was regarded as a new hallmark of cancer cells, such as colorectal, gastric, breast, prostate, hepatocellular, as well as renal cell carcinoma and glioblastoma. In addition,

high LD accumulation is increasingly recognized as a predictor of aggressive cancer [14]. It has been proven that higher amounts of triglycerides and cholesterol stored in LDs are correlated with poorer prognosis and shorter disease-free survival for many types of cancer. Moreover, undifferentiated stem cells also contain higher amounts of lipid droplets than their differentiated counterparts. Given that lipids play a role in regulating cellular processes and can influence a wide range of tumorigenic steps in GC development, progression, and metastasis, there is significant research interest in the development of therapies that target lipid metabolism.

In this context, we integrated clinical, lipidomics, and transcriptomics data and revealed that GC exhibited a reprogramming of fatty acid metabolism by altered lipid levels and abnormal lipid metabolism-associated gene models. The possible associations of lipid metabolism mechanisms with GC progression were first described in this paper, focusing on investigating candidate signature genes and biological events that occur during progression. Several studies have been conducted to explain this phenomenon. Tumour cells acquire diet-derived fatty acids (FAs) from the blood, which are subsequently used to produce more energy for cancer cell proliferation and growth. In the case of cancer cell nutrient deprivation, fatty acids released from LDs are used instead for energy production via mitochondrial β-oxidation and the Krebs cycle [15]. Moreover, the extracellular FAs provide a compensatory mechanism for cancer cells under conditions of metabolic stress [16]. LD accumulation may also impair drug-induced apoptosis and immunogenic cell death, resulting in chemotherapy resistance in cancer cells. Lipolysis enzymes can bind to the cell luminal surface to remodel plasma membranes, potentially enhancing membrane saturation. Saturated membranes may function in making cancer cells less susceptible to free radicals and thereby reducing the efficacy of certain chemotherapeutic agents [17].

*ACLY* is an upstream enzyme that connects carbohydrate metabolism with lipid metabolism, generating acetyl-CoA from citrate to supply oxaloacetate and acetyl on the cytosolic side, along with allowing acetylation of histone substrates [18]. *ACLY* is reportedly overexpressed in many types of cancers, including osteosarcoma, cervical cancer, prostate cancer, lung cancer, hepatic, and colorectal cancers [19], and can contribute to tumour progression. Our data showed that *ACLY* promoted de novo FA synthesis in GC cells by upregulating the lipogenic enzymes *ACACA1, FASN,* and *SCD1*, further providing evidence for the oncogenic role of dysregulated lipogenic enzymes (Fig. 6). *ACLY* is upstream of *HMGCR* and is an important enzyme in the cholesterol biosynthetic pathway, which regulates multiple downstream pathways of lipid metabolism. Most importantly, *ACLY* catalyses the conversion of citric acid to oxaloacetate and acetyl-CoA, which subsequently upregulates key lipid metabolism enzymes [20, 21]. *ACLY* regulates the expression of the ATP-binding cassette sterol transporter *ABCG5/8* [22], which catalyses the final step in reverse cholesterol transport. The *ACLY* gene promoter contains a sterol response element, whose expression is regulated by sterol regulatory element binding protein-1α *(SREBP-1α)* [19, 23]. In addition to downregulation of lipid levels, *SREBP-1a* displays tumour suppressive effects by attenuating aerobic glycolysis in tumour cells in vitro, reducing tumour growth, and inducing differentiation in vivo [24, 25]. In this study, we found that *ACLY* robustly promote lipid metabolism in GC cells, which further facilitate tumor cell migration and transwell (Fig. 6).

*ACLY* is involved in the chemotherapeutic response of GC. For example, *ACLY* is involved in the AMPK pathway, which plays a key role in mediating chemoresistance in breast cancer [26]. *ACLY* also plays a significant role in the AKT signaling pathway, promoting the survival of drug-resistant colorectal cancer cells [27]. *ACLY* inhibitors have shown clinical efficacy in the treatment of dyslipidaemia and other cardiovascular disorders when used as monotherapy or combination therapy with other lipid-modulating drugs. Studies have shown that *ACLY* inhibitors have various pharmacological effects, specifically in the reduction of non-HDL-C, TG, and insulin levels, as well as increasing plasma β-hydroxybutyrate levels.

Hyperactive of *mTORC1* signaling is a major cause of human tumors, and *mTORC1* has been investigated as a potential target for cancer therapy. Rapamycin analogues (rapalogs) such as everolimus and temsirolimus have been approved for use in some advanced carcinomas. Further research on the regulation of *mTORC1* is of considerable biological and clinical interest. Since the *AKT/mTOR* pathway is known to play a central role in the regulation of cell lipid metabolism, we hypothesised that blocking *ACLY* will reduce lipid synthesis and subsequently prevent tumour cell resistance to TKI therapy, enhancing its antitumor efficacy. *ACLY* can also affect nuclear receptors, promoting the transcription of the ABC family transporter *ABCB1/ABCG2,* which is involved in the development of multi-drug resistance [28]. Mehdizadeh et al. reported that changes in FA distribution in gastrointestinal cancer cells are associated with adverse side effects of conventional chemotherapy.

## Supplementary Information

**Additional file 1: Table S1.** The detailed information of GEO chips.

**Additional file 2: Fig. S1.** Flow chart of the experimental design and main process. **Fig. S2.** Kaplan-Meier curves for GC patients stratified by lipid factors. (A) Kaplan-Meier analysis of Progression-free survival (PFS) of Cholesterol, Triglyceride, HDL and LDL;(B) Nomogram developed by integrating metabolic syndrome and clinical pathological parameters for predicting 1-, 3-, 5-year PFS survival of GC patients; (C) Calibration curve for risk of 1-, 3-, 5-year PFS survival of metabolic syndrome.

## Data Availability

The datasets used and/or analyzed during the current study are available from the TCGA (https://www.cancer.gov/about-nci) and GEO (GSE15459, GSE26253, GSE62254 and GSE84437, https:// www.ncbi.nlm.nih.gov) database and the First Affiliated Hospital of Wenzhou Medical University.
