# Supplementary material for: Integration of clinical and transcriptomics reveals programming of the lipid metabolism in gastric cancer
Source: BMC Cancer. 2022 Sep 6;22:955. doi: 10.1186/s12885-022-10017-4 (PMC9446547; doi:10.1186/s12885-022-10017-4)
Supplement: Supplementary file 1 — Additional file 1: Table S1. The detailed information of GEO chips. [file 12885_2022_10017_MOESM1_ESM.docx]

Table S1 The detailed information of GEO chips

| Accession | Platform | Number of normal samples | Number of cancer samples |
| --- | --- | --- | --- |
| GSE2685 | GPL80 | 8 | 22 |
| GSE13911 | GPL570 | 31 | 38 |
| GSE26988 | GPL6947 | 12 | 96 |
| GSE29272 | GPL96 | 134 | 134 |
| GSE37023 | GPL96 | 40 | 114 |
| GSE54129 | GPL570 | 21 | 111 |
| GSE66229 | GPL570 | 100 | 300 |
| GSE12369 | GPL15207 | 6 | 36 |
| GSE26942 | GPL6947 | 12 | 205 |
| GSE84787 | GPL17077 | 10 | 10 |
| GSE65801 | GPL14500 | 32 | 32 |
| GSE79973 | GPL570 | 10 | 10 |
| TCGA | TCGA | 32 | 380 |
| Total | 1958 | 448 | 1488 |
