# Supplementary material for: Integration of clinical and transcriptomics reveals programming of the lipid metabolism in gastric cancer
Source: BMC Cancer. 2022 Sep 6;22:955. doi: 10.1186/s12885-022-10017-4 (PMC9446547; doi:10.1186/s12885-022-10017-4)
Supplement: Supplementary file 2 — Additional file 2: Fig. S1. Flow chart of the experimental design and main process. Fig. S2. Kaplan-Meier curves for GC patients stratified by lipid factors. (A) Kaplan-Meier analysis of Progression-free survival (PFS) of Cholesterol, Triglyceride, HDL and LDL;(B) Nomogram developed by integrating metabolic syndrome and clinical pathological parameters for predicting 1-, 3-, 5-year PFS survival of GC patients; (C) Calibration curve for risk of 1-, 3-, 5-year PFS survival of metabolic syndrome. [file 12885_2022_10017_MOESM2_ESM.zip › supplemental figure legend.docx]

**Supplemental material**

**Figure S1** Flow chart of the experimental design and main process.

**Figure S2** Kaplan-Meier curves for GC patients stratified by lipid factors. (A) Kaplan-Meier analysis of Progression-free survival (PFS) of Cholesterol, Triglyceride, HDL and LDL;(B)Nomogram developed by integrating metabolic syndrome and clinical pathological parameters for predicting 1-, 3-, 5-year PFS survival of GC patients; (C)Calibration curve for risk of 1-, 3-, 5-year PFS survival of metabolic syndrome;
